# Physical Properties and Precipitate Microstructures of Cu-Hf Alloys at Different Processing Stages

**DOI:** 10.1155/2018/3653987

**Published:** 2018-12-17

**Authors:** Mingmao Li, Leqing Zhang, Mingbiao Zhu, Hang Wang, Haigen Wei

**Affiliations:** ^1^National Engineering Research Centre for Copper Smelting and Manufacturing, Institute of Engineering Research, Ganzhou, Jiangxi 341000, China; ^2^School of Materials Science and Engineering, Jiangxi University of Science and Technology, Ganzhou, Jiangxi 341000, China

## Abstract

The microstructural evolution and hardness and physical properties of a Cu-Hf alloy at the different processing stages were investigated using hardness, conductivity and tensile measurements, metallographic microscopy, scanning electron microscopy, and transmission electron microscopy. The results reveal that the electrical conductivity of these alloys was above 80% IACS after aging at 450°C, and the hardness and conductivity of the Cu-0.9Hf alloy were 180 HV0.5 and 80% IACS, respectively. The softening temperature of the Cu-0.15Hf alloy is 525°C, and the softening temperature of Cu-0.4Hf and Cu-0.9Hf alloys is 550°C. The precipitated Hf-containing phase exhibited a short rod-like structure, the size of which increased with aging time at a slow rate and resulted in the size of ~20 nm after aging at 450°C for 300 min.

## 1. Introduction

Copper has excellent electrical and thermal conductivity, but poor strength and heat resistance limit its applications in a wide range of areas. Therefore, copper alloys are prepared by adding metallic or nonmetallic elements into copper to attain desirable strength and heat resistance [1]. Among copper alloys, many binary alloys, such as Cu-Ag [2, 3], Cu-Zr [4, 5], Cu-Sn [6, 7], Cu-Mg [8, 9], and Cu-Cr [10–12] have been widely investigated and utilized in a variety of electrical and electronic applications, including electrical energy transmission. However, the addition of alloying elements has shown little influence on the electrical conductivity, although some of these can improve the mechanical strength, recrystallization temperature, and wear resistance.

**Figure 1:**
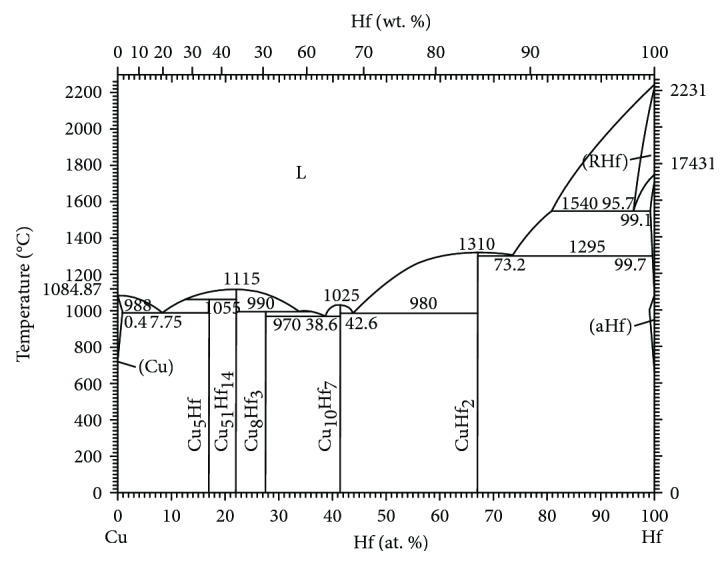
Binary phase diagram of the Cu-Hf alloy.

Hafnium (Hf) has exhibited interesting characteristics, such as plastic behavior, ease of processing, excellent temperature, and corrosion resistance, which make it a desirable material in atomic reactors and nickel-based superalloys [13–15]. From a structural and performance viewpoint, Hf is similar to Zr and it can increase the recrystallization temperature and heat resistance of copper. In addition, the high electrical conductivity is maintained and mechanical strength is improved due to Hf addition [16]. Moreover, a precipitation strengthening effect has also been observed in Hf-Cu alloys. Furthermore, the solubility of Hf in copper is higher than Zr (from the binary phase diagram of the Cu-Hf alloy (Figure 1), we can know that the maximum solubility of Hf in Cu is 0.4 at.% [17], while Zr is only 0.12 at.%) at eutectic temperature, which suggests further performance improvement [18]. Shangina et al. have studied the properties, microstructure, and heat resistance of the Cu-0.9Hf alloy after aging under high-pressure torsion and equal channel angular extrusion. They have also compared the dislocation density after different processes [19, 20].

In this work, the influence of Hf addition on the mechanical and physical properties of copper and the microstructural evolution of the Cu-Hf alloy, under hot rolling, solid solution, cold rolling and aging process, have been studied. Moreover, we have also investigated the morphology and size of the precipitates in the aging process.

## 2. Experimental

The experiment was carried out by using an oxygen-free copper rod and a Cu-8 wt.% Hf intermediate alloy as raw materials. The alloy was prepared in a vacuum induction furnace under argon flow as a shielding gas; then, it was cast into a cylindrical ingot with a diameter of 30 mm under vacuum.

According to the maximum solid solubility of Hf in Cu in Figure 1, we designed three sets of Cu-Hf alloys with different Hf contents, and the composition of the alloys were measured by inductively coupled plasma optical emission spectrometry (ICP), as shown in Table 1. The samples were sectioned into ingots with a dimension of Φ30 mm × 20 mm and hot rolled. The rolling temperature was 900°C, and the deformation was 76.5%. Subsequently, the hot-rolled samples were solid-solution treated in a box-type resistance furnace and the Cu-Hf alloy solid solution process was carried out at 950°C for 1 h. The samples were quenched in water after solid-solution treatment. After solid-solution treatment, the upper and lower milling surfaces were ~4 mm thick, which were then cold-rolled into a sheet of ~2 mm thickness with a deformation of 50%. Finally, the aging treatment was carried out and the Cu-Hf alloy was aged at 400°C and 450°C for 10, 30, 60, 120, 180, 240, and 300 min.

The electrical conductivity, Vickers hardness, and tensile strength of each alloy were measured by using an eddy current conductivity meter (Sigma 2008 B/C), a Vickers hardness tester (FM-700 type, Future-Tech Corp., Tokyo Japan) (HV 0.5, test force 4.903 N), and a universal testing machine (UTM5105X, Shenzhen Sunthink), respectively. The microstructure of the alloy was observed by an optical microscope (OM, Axioskop 2, Zeiss, Germany) and scanning electron microscopy (SEM, MLA 650F, FEI, USA). The morphology and crystal structure of the precipitated phase were further analyzed by transmission electron microscopy (TEM, Tecnai G2 20, FEI, USA) and selected area electron diffraction (SAED) patterns. TEM samples were prepared by sectioning alloy blocks into a thin sheet of 60 *μ*m and punching in the shape of a round foil with 3 mm diameter. The round foil was electrolytically polished at 40 V in a double jet electrolyzer, and sample temperature was maintained at −40°C. The electropolishing solution was a mixture of nitric acid and methanol (1 : 4).

The change in hardness values with respect to temperature has been assessed by annealing Cu-Hf alloys at different temperatures for 1 hour. Furthermore, the softening temperature of each alloy was obtained.

## 3. Results

### 3.1. Microhardness and Physical Properties

**Figure 2:**
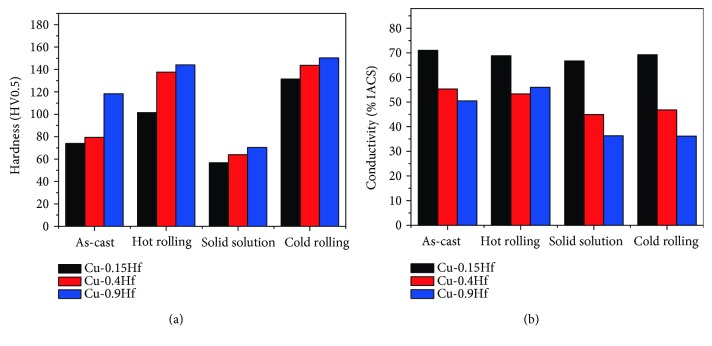
Hardness and conductivity of as-cast, hot-rolled, solid-solution-treated and cold-rolled Cu-Hf alloys before aging: (a) hardness and (b) conductivity.

Figure 2 presents the hardness and electrical conductivity of as-cast, hot-rolled, solid-solution-treated and cold-rolled Cu-0.15Hf, Cu-0.4Hf, and Cu-0.9Hf alloys before aging treatment. For the as-cast alloy state, the hardness of the alloy increased with the increase of Hf content. For instance, Cu-0.9Hf exhibited the hardness value of 118.2 HV0.5, which is 2 times higher than the Cu-0.15Hf alloy. However, the conductivity has shown an opposite trend and decreased with the addition of Hf. Compared with pure copper, the conductivity of Cu-0.9Hf was only 50.6% IACS. In hot-rolled alloys, the hardness has been improved and the variation is quite large. However, conductivity exhibited a little change with respect to Hf addition. After solid-solution treatment, the hardness of the alloys sharply decreased due to recrystallization, grain growth, and dissolution of the solute into the copper matrix. Similarly, the electrical conductivity has shown an inverse relationship with Hf content. For instance, the conductivity of Cu-0.4Hf was 44.9% IACS and the conductivity of Cu-0.9Hf dropped to 36.3% IACS. In cold-rolled Cu-Hf alloys, the hardness of the alloy increased to a higher level and Cu-0.15Hf, Cu-0.4Hf, and Cu-0.9Hf alloys exhibited hardness values of 131.5 HV0.5, 143.8 HV0.5, and 150.3 HV0.5, respectively. However, the conductivity has shown a small change in cold-rolled alloys as well.

**Figure 3:**
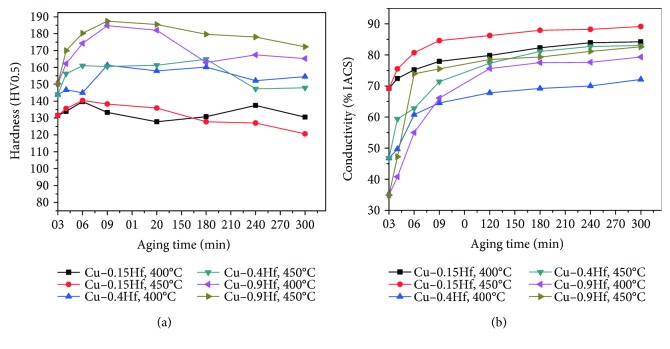
The hardness and conductivity of the Cu-Hf alloy after aging: (a) hardness and (b) conductivity.

Figure 3 presents the hardness and electrical conductivity of the Cu-0.15Hf, Cu-0.4Hf, and Cu-0.9Hf alloys after aging treatment, which has been carried out for different temperatures and times. Comparing the properties of the alloy after aging at 400°C and 450°C, it can be concluded that the optimum aging temperature for the three alloys should be 450°C. The peak aging of Cu-0.15Hf, Cu-0.4Hf, and Cu-0.9Hf alloys can be obtained at 30 min, 30 min, and 60 min, respectively. In addition, the hardness values of 140.4 HV0.5, 161.0 HV0.5, and 187.4 HV0.5 and conductivity values of 80.0% IACS, 65.0% IACS, and 75.5% IACS have been achieved for Cu-0.15Hf, Cu-0.4Hf, and Cu-0.9Hf alloys, respectively.

**Figure 4:**
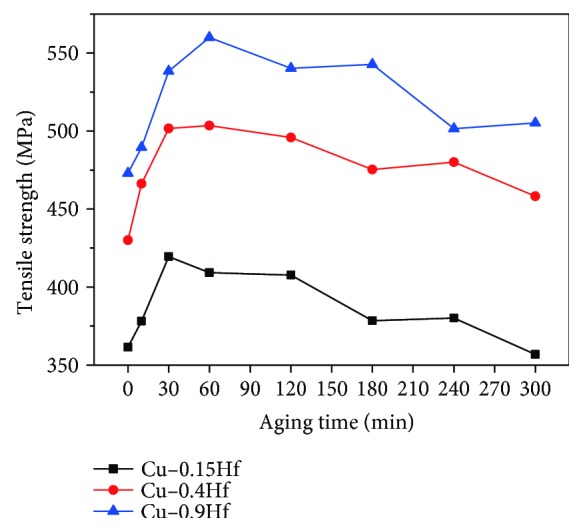
Tensile strength of Cu-Hf alloys after aging at 500°C.

Figure 4 shows the tensile strength of Cu-0.15Hf, Cu-0.4Hf, and Cu-0.9Hf alloys after aging at 500°C. It can be observed that the strength of Cu-Hf alloys rapidly increases with time in the initial stage and then gradually decreases, exhibiting a regularity that is consistent with changes in hardness. However, the conversion between the tensile strength and the hardness of the alloy does not correspond well, since the measured value of tensile strength is less than the tensile strength converted from hardness.

**Figure 5:**
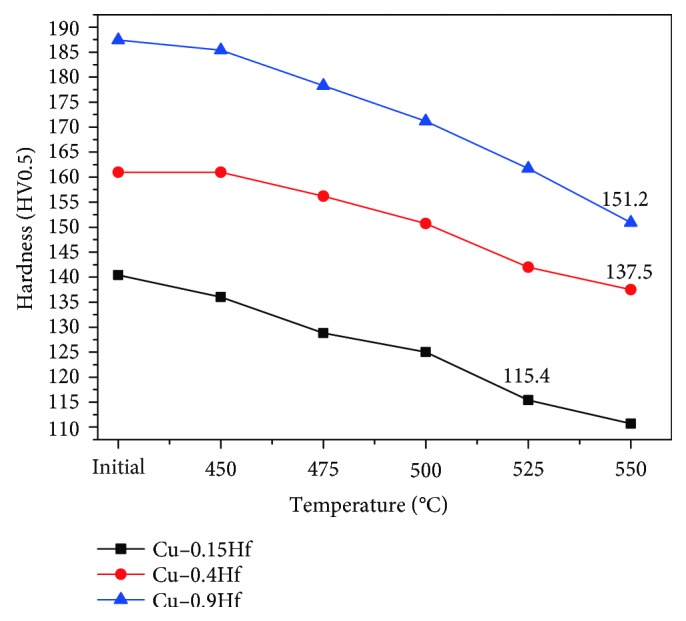
Relationship between hardness and annealing temperature of Cu-Hf alloys.

Figure 5 shows the change in hardness of Cu-0.15Hf, Cu-0.4Hf, and Cu-0.9Hf alloys after annealing at different temperatures. Owing to recrystallization and grain growth, the hardness of the alloys has been decreased. According to the GB/T 33370-2016 method, the softening temperature can be defined based on the hardness values and 80% of the original hardness corresponds to the softening temperature of the alloy. It can be concluded that the softening temperature of Cu-0.15Hf, Cu-0.4Hf, and Cu-0.9Hf alloys is 525°C, 550°C, and 550°C, respectively.

### 3.2. Morphology of Microstructures

#### 3.2.1. As-Cast Cu-Hf Alloys

**Figure 6:**
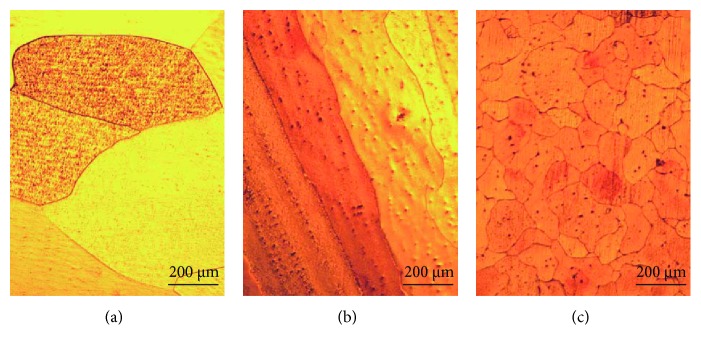
The optical micrographs of as-cast Cu-Hf alloys: (a) Cu-0.15Hf, (b) Cu-0.4Hf, and (c) Cu-0.9Hf.

Figure 6 shows the optical micrographs of as-cast Cu-0.15Hf, Cu-0.4Hf, and Cu-0.9Hf alloys. It can be clearly seen that all three alloys exhibited typical as-cast structures, and Cu-0.15Hf and Cu-0.4Hf alloys have shown coarser grains (>500 *μ*m) than Cu-0.9Hf (<200 *μ*m). This implies that Hf addition has some sort of grain refinement effect on the as-cast microstructure of Cu after a threshold level.

**Figure 7:**
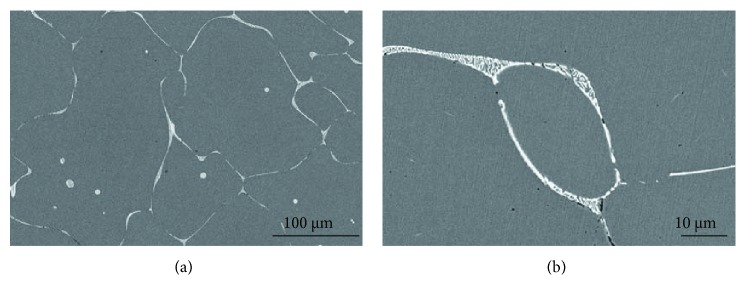
SEM images of the as-cast Cu-0.9Hf alloy at different magnifications.

**Figure 8:**
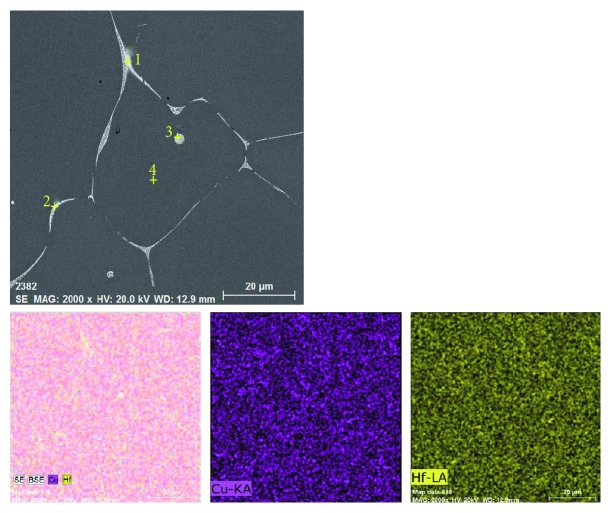
Elemental distribution of the as-cast Cu-0.9Hf alloy.

Figure 7 presents the SEM images of the as-cast Cu-0.9Hf alloy. One should note that the maximum solubility of elemental Hf in copper is 0.4 at.%; however, the actual solubility is lower than 0.4 at.% at a low temperature. Hence, during ingot cooling, Hf has been desolvated and a primary phase has been formed containing Hf. It can also be seen in Figure 7(a) that most of the white precipitates have been distributed along the grain boundaries. In addition, the precipitate distribution is dense and uniform. However, a small portion of the primary phase is distributed in the grains and has a circular morphology.  Figure 7(b) is a high-magnification SEM image of the same alloy and exhibits a sheet-like eutectic structure, which is consistent with the Cu-Hf binary phase diagram.  Figure 8 and Table 2 show the element distribution diagram of the as-cast Cu-0.9Hf alloy and the distribution point analysis of the alloy composition, respectively. We have not observed the Hf aggregation in elemental distribution maps, but the composition distribution points revealed that the Hf element is present in the white eutectic structure instead of the Cu matrix.

#### 3.2.2. Hot-Rolled Alloys

**Figure 9:**
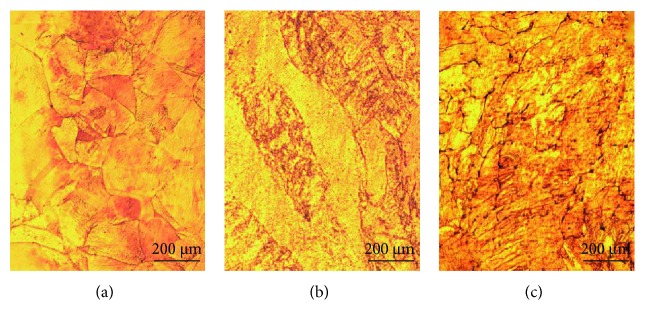
Optical micrographs of the hot-rolled Cu-Hf alloys: (a) Cu-0.15Hf, (b) Cu-0.4Hf, and (c) Cu-0.9Hf.

Figure 9 shows the optical micrograph of hot-rolled Cu-0.15Hf, Cu-0.4Hf, and Cu-0.9Hf alloys. Compared with as-cast alloys, the grain size of the Cu-0.15Hf alloy decreased and the grain size of the Cu-0.4Hf and Cu-0.9Hf alloys remained the same.  Figure 9(b) shows that the grains have been elongated in the rolling direction and exhibit a warm deformation structure, indicating that the finishing temperature is still not higher than the dynamic recrystallization temperature, especially for Cu-0.9Hf.

#### 3.2.3. Solid-Solution-Treated Cu-Hf Alloys

**Figure 10:**
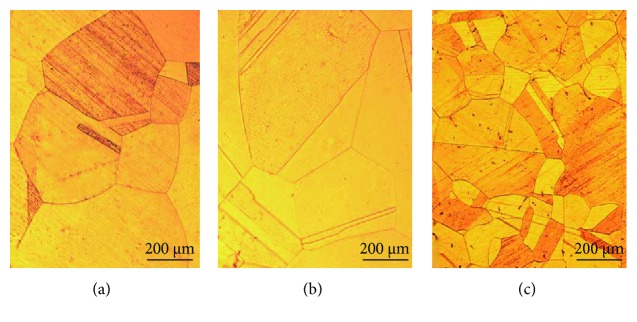
Optical micrograph of the solid-solution-treated Cu-Hf alloy: (a) Cu-0.15Hf, (b) Cu-0.4Hf, and (c) Cu-0.9Hf.

**Figure 11:**
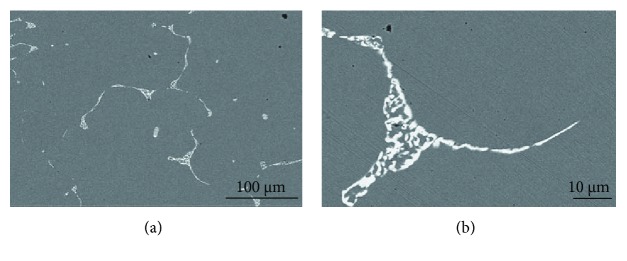
The SEM images of the solid-solution-treated Cu-0.9Hf alloy at different magnifications.

Figure 10 shows the optical micrographs of solid-solution-treated Cu-0.15Hf, Cu-0.4Hf, and Cu-0.9Hf alloys. The three alloys are completely recrystallized and the grains have grown to a certain extent. Moreover, we have observed annealing twins after solid-solution treatment and Cu-0.9Hf has exhibited smaller twin crystals than the other two alloys.  Figure 11 presents the SEM images of the Cu-0.9Hf alloy after solid-solution treatment. It can be seen from Figure 11(a) that the white sheet eutectic structure is no longer continuous and the edges are smoothened.  Figure 11(b) shows that the sheet-like features gradually disappeared and the microstructure became smaller and round-shaped, which indicates that solid-solution treatment at 950°C dissolved the primary phase into the copper matrix.

#### 3.2.4. Cold-Rolled Cu-Hf Alloys

**Figure 12:**
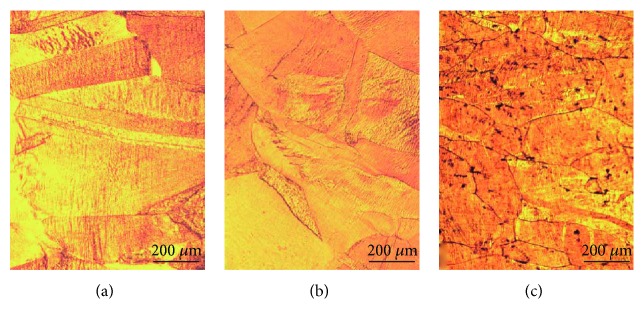
Optical micrographs of cold-rolled Cu-Hf alloys: (a) Cu-0.15Hf, (b) Cu-0.4Hf, and (c) Cu-0.9Hf.

Figure 12 shows the optical micrographs of Cu-0.15Hf, Cu-0.4Hf, and Cu-0.9Hf alloys after cold rolling. The three alloys have shown an elongated structure compared to solid-solution-treated alloys. Moreover, a certain directionality has been observed and the grain boundaries became blurred due to cold deformation. Cold deformation generates a large number of dislocations and improves the mechanical properties of the alloy. In addition, deformation energy gets stored in the alloy and a strengthening phase can be quickly precipitated during aging.

#### 3.2.5. Aging of Cu-Hf Alloys

**Figure 13:**
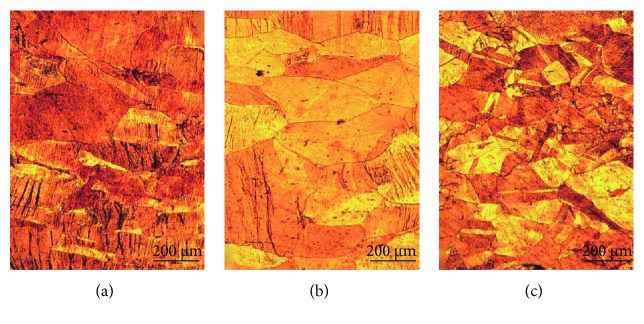
Optical micrographs of the Cu-0.9Hf alloy after aging at 450°C: (a) 10 min, (b) 60 min, and (c) 300 min.

**Figure 14:**
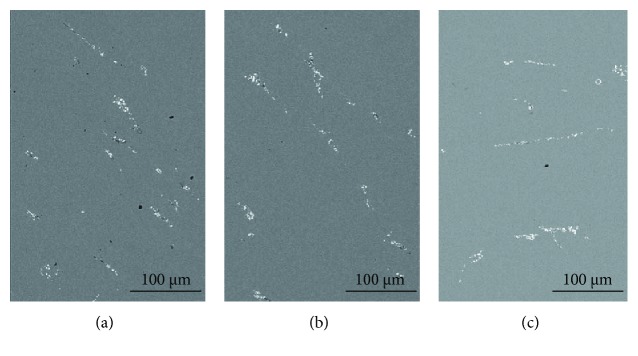
SEM images of the Cu-0.9Hf alloy after aging at 450°C: (a) 10 min, (b) 60 min, and (c) 300 min.

In order to understand the influence of Hf addition on the heat resistance of Cu, we have selected the Cu-0.9Hf alloy for further experiments and the three aging states, i.e., 10 min, 60 min, and 300 min, were used. Figures 13 and 14 present the optical micrograph and SEM images of the Cu-0.9Hf alloy after aging at 450°C for 10 min, 60 min, and 300 min, respectively. The optical micrographs clearly demonstrate that cold rolling induced a deformed microstructure after aging for 10 min and 60 min. However, after 300 min, the recrystallization has been completed and twins have appeared. SEM images in Figure 14 exhibit the banded structure, which is distributed in a certain direction due to cold rolling. The distribution of these primary phases along the rolling direction indicate that the aging process does not cause excessive redistribution.

**Figure 15:**
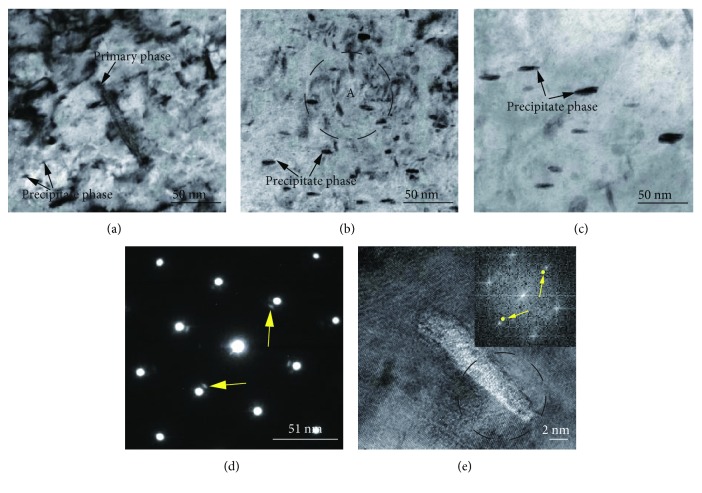
Bright-field TEM images of the Cu-0.9Hf alloy after aging at 450°C: (a) 10 min, (b) 60 min, and (c) 300 min. (d) SAED pattern from a selected area, marked as A in (b) and (e) high-resolution TEM images and FFT pattern from the precipitated phase.

In order to assess the type of precipitation phase and quantify it with respect to aging time, we have observed the morphology of the Cu-0.9Hf alloy after aging at 450°C by using transmission electron microscopy (TEM). We have studied the peak aging samples and corresponding SAED patterns, and high-resolution bright-field images were obtained. TEM observations clearly demonstrated the precipitation trend with respect to aging time.  Figure 15 shows the bright-field images and corresponding SAED patterns of the Cu-0.9Hf alloy after aging at 450°C. Figures 15(a)–15(c) show that the precipitated particles have a rod-like structure, which is uniformly distributed in the matrix. The size of the rods has shown a direct relationship with aging time, and sizes of 3–5 nm, 10 nm, and 20 nm have been attained after aging for 10, 60, and 300 min, respectively. In Figure 15(a), we have also observed coarse primary precipitates, which are larger than 100 nm.  Figure 15(d) presents the SAED pattern of the area, marked as A. It can be clearly observed that there are other weak areas around the [011] Cu spot. The fast Fourier transform (FFT) of high-resolution bright-field image of the precipitated phase resulted in additional diffraction spots, which are at the same position as in Figures 15(d) and 15(e). The spots are marked by yellow arrows in Figure 15(e).

**Figure 16:**
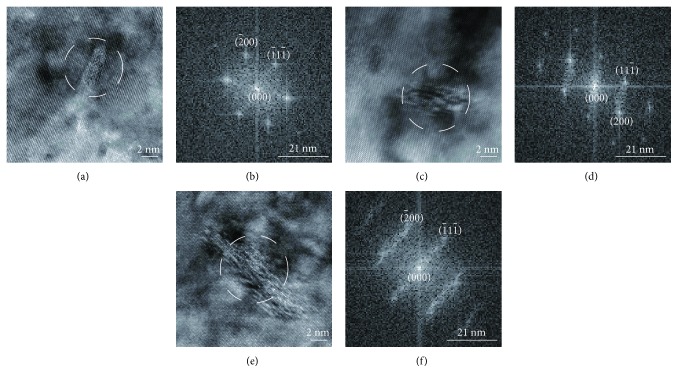
High-resolution TEM images and corresponding FFT patterns of the Cu-0.9Hf alloy, aged at 450°C for different times: (a, b) 10 min, (c, d) 60 min, and (e, f) 300 min.

Figure 16 shows the high-resolution TEM images and FFT of the Cu-0.9Hf alloy after aging for different times. Figures 16(a), 16(c), and 16(e) clearly demonstrate that the size of the precipitated particles gradually increased with aging time. Figures 16(b), 16(d), and 16(f) present the corresponding FFT patterns and indicate the presence of FCC Cu after aging for 10 min. The FCC structure of Cu is also present after further aging; however, other spots gradually appeared in FFT patterns, which correspond to the precipitated phase.

## 4. Discussion

This study shows that Hf addition influences the physical and mechanical properties of Cu. It has been found that the addition of the Hf element improves the hardness of the as-cast Cu-Hf alloy, and a direct relationship exists between hardness and Hf content. For instance, the hardness of the Cu-0.9Hf alloy reached up to 120 HV0.5, which is two times higher than the Cu-0.15Hf alloy. Also, Hf addition influenced the conductivity of the Cu-Hf alloy. Furthermore, hot rolling removed some of the alloy defects and made it denser, which improves the mechanical properties of the alloy and reduces the number of passes after the rolling. After solid-solution treatment, the cold rolling rapidly increased the hardness of the alloy to a higher level, but the influence on electrical conductivity remained negligible. In addition, it also provides deformation energy storage for the aging process, which causes the precipitation of the secondary phase and influences the recrystallization temperature of the alloy. On the other hand, the aging treatment desolvates the solute from the matrix and results in a precipitated phase, which enhances the hardness and electrical conductivity of the alloy. It is worth noting that the hardness of Cu-0.9Hf reached up to 180 HV0.5, and conductivity attained a value of 80% IACS after aging treatment. The results show that the influence of the Hf element on Cu conductivity becomes smaller due to reasonable deformation and the heat treatment process, and ideal comprehensive properties can be achieved. The addition of Hf can greatly increase the softening temperature of Cu. When the Hf content is more than 0.4 wt. %, the softening temperature reached 550°C.

Furthermore, Hf addition has shown a certain grain refinement effect on the microstructure of as-cast Cu after a threshold value. Although the deformation and heat treatment have a significant influence on the alloy microstructure, the role of Hf addition cannot be neglected. In terms of grain size, although the recrystallization and grain growth occurred during solid-solution treatment, the grain size of the Cu-0.9Hf alloy is lower than the alloy with a lower Hf content. Similarly, Hf addition influenced the recrystallization of the alloy aging process and effectively increased the recrystallization temperature of Cu.

The study also showed that the size of the precipitated phase increased with time, but the rate was very slow. That is why the Cu-0.9Hf alloy maintained the highest hardness among tested alloys. In addition to Cu FCC spots, we have also observed some weak spots in the SAED and FFT patterns at the same position, which implies the presence of a secondary precipitated phase.

As mentioned above, Hf addition into Cu inhibits recrystallization and improves heat resistance and this effect is more obvious as the Hf content increases. At the same time, an Hf-containing phase is formed, which is uniformly distributed and hinders the movement of grain boundaries and pinning dislocations. The presence of the Hf-containing secondary phase also contributes to the hardness of the alloy. Under the deformation and heat treatment process, the solute is desolvated during the aging process and forms a fine precipitated phase, and the scattering effect on electrons becomes very small, which enhances the electrical conductivity of the Cu-Hf alloy. In summary, Cu-Hf alloys offer impressive electrical and mechanical properties, which make them desirable for a wide range of applications.

## 5. Conclusions

The influence of Hf addition on the microstructure, mechanical properties, and electrical properties of Cu has been investigated by optical microscopy, scanning electron microscopy (SEM), and transmission electron microscopy (TEM). The mechanism of improved thermal stability of alloys due to Hf addition has been presented. In summary, the following conclusions can be drawn from this study:

1. From the viewpoint of mechanical properties, the Cu-Hf alloy has shown optimal performance at an aging temperature of 450°C. The softening temperature of the Cu-0.15Hf alloy was 525°C, and the softening temperature of Cu-0.4Hf and Cu-0.9Hf alloys were 550° C. The hardness of Cu-0.15Hf and Cu-0.4Hf alloys, after alloying, reached up to 140 HV0.5 and 160 HV0.5, respectively. Similarly, the electrical conductivity values reached above 80% IACS. The hardness and conductivity of the Cu-0.9Hf alloy, after aging, were 180 HV0.5 and 80% IACS, respectively.
2. The Hf addition increased the hardness of the as-cast alloy. Furthermore, the deformation and heat treatment processes hindered the grain growth and improved the heat resistance of the alloy. These properties have shown a direct relationship with the Hf content.
3. After aging treatment, the precipitated phase of the Cu-Hf alloy exhibited a rod-like structure, with a particle size of ~20 nm, at 450°C for 300 min. However, the growth rate has exhibited a little influence on the hardness, that is, this alloy is not susceptible to overaging.

## Figures and Tables

**Table 1:** The compositions of Cu-Hf alloys, measured by ICP (mass fraction, %).

Alloy number	Hf	Cu
1#	0.15	Bal.
2#	0.4	Bal.
3#	0.9	Bal.

**Table 2:** Point composition analysis of the as-cast alloy (atomic fraction, %).

Alloys	Test position	Hf	Cu
Cu-0.9Hf	1	8.04	91.96
	2	10.19	89.81
	3	3.07	96.93
	4	0.00	100.00

## Data Availability

Our experimental data and conclusions come from repeated experiments, and the same results can be obtained according to the experimental process in the manuscript.
